# Supplementary material for: Causal Relationship between Aging and Anorexia Nervosa: A White-Matter-Microstructure-Mediated Mendelian Randomization Analysis
Source: Biomedicines. 2024 Aug 16;12(8):1874. doi: 10.3390/biomedicines12081874 (PMC11351342; doi:10.3390/biomedicines12081874)
Supplement: Supplementary file 1 [file biomedicines-12-01874-s001.zip › Supplementary Figure/Figure S4/Singe SNP plot of MR analysis for TL to ubm-b-1770.pdf]

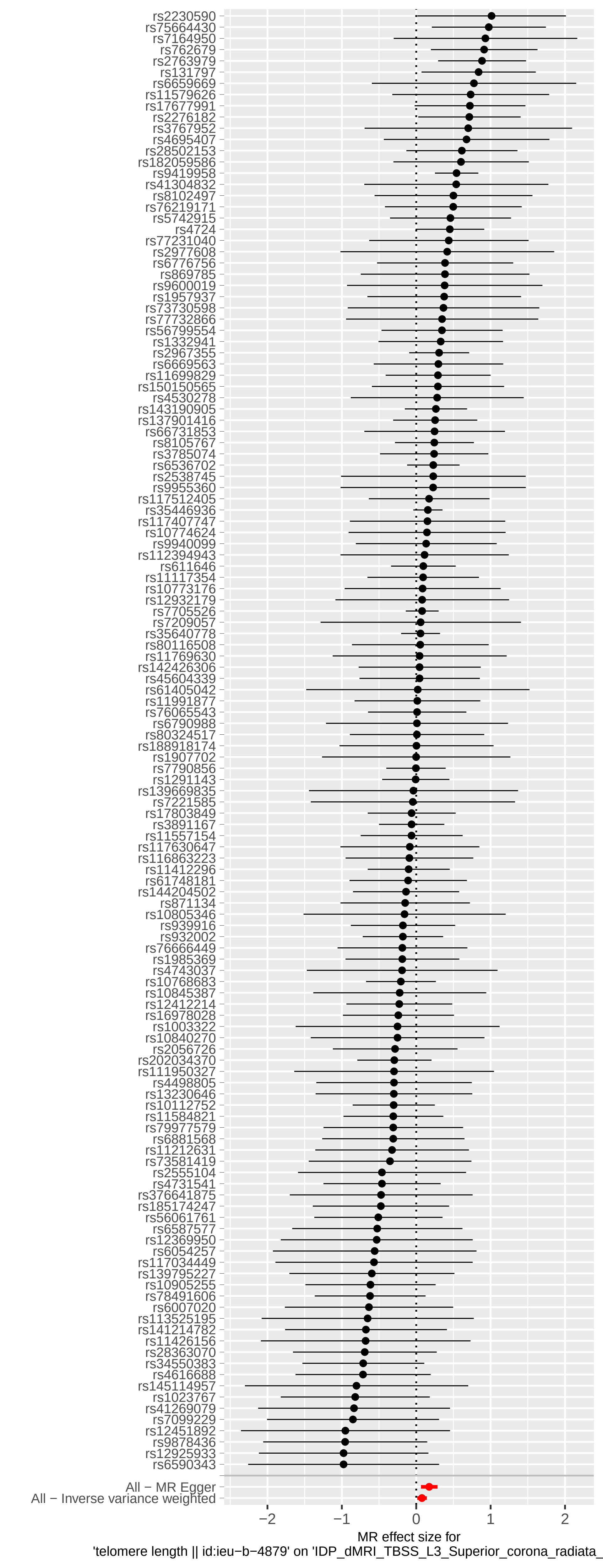
rs2230590
rs75664430
rs7164950
rs762679
rs2763979
rs131797
rs6659669
rs11579626
rs17677991
rs2276182
rs3767952
rs4695407
rs28502153
rs182059586
rs9419958
rs41304832
rs8102497
rs76219171
rs5742915
rs4724
rs77231040
rs2977608
rs6776756
rs869785
rs9600019
rs1957937
rs73730598
rs77732866
rs56799554
rs1332941
rs2967355
rs6669563
rs11699829
rs150150565
rs4530278
rs143190905
rs137901416
rs66731853
rs8105767
rs3785074
rs6536702
rs2538745
rs9955360
rs117512405
rs35446936
rs117407747
rs10774624
rs9940099
rs112394943
rs611646
rs11117354
rs10773176
rs12932179
rs7705526
rs7209057
rs35640778
rs80116508
rs11769630
rs142426306
rs45604339
rs61405042
rs11991877
rs76065543
rs6790988
rs80324517
rs188918174
rs1907702
rs7790856
rs1291143
rs139669835
rs7221585
rs17803849
rs3891167
rs11557154
rs117630647
rs116863223
rs11412296
rs61748181
rs144204502
rs871134
rs10805346
rs939916
rs932002
rs76666449
rs1985369
rs4743037
rs10768683
rs10845387
rs12412214
rs16978028
rs1003322
rs10840270
rs2056726
rs202034370
rs111950327
rs4498805
rs13230646
rs10112752
rs11584821
rs79977579
rs6881568
rs11212631
rs73581419
rs2555104
rs4731541
rs376641875
rs185174247
rs56061761
rs6587577
rs12369950
rs6054257
rs117034449
rs139795227
rs10905255
rs78491606
rs6007020
rs113525195
rs141214782
rs11426156
rs28363070
rs34550383
rs4616688
rs145114957
rs1023767
rs41269079
rs7099229
rs12451892
rs9878436
rs12925933
rs6590343
All − MR Egger
All − Inverse variance weighted
−2
−1
0
1
2
MR effect size for
'telomere length || id:ieu−b−4879' on 'IDP_dMRI_TBSS_L3_Superior_corona_radiata_
